# Nomophobia in Lebanon: Scale validation and association with psychological aspects

**DOI:** 10.1371/journal.pone.0249890

**Published:** 2021-04-20

**Authors:** Youssef Farchakh, Rabih Hallit, Marwan Akel, Clarissa Chalhoub, Maria Hachem, Souheil Hallit, Sahar Obeid

**Affiliations:** 1 Faculty of Medicine and Medical Sciences, Holy Spirit University of Kaslik (USEK), Jounieh, Lebanon; 2 INSPECT-LB: National Institute of Public Health, Clinical Epidemiology and Toxicology, Beirut, Lebanon; 3 School of Pharmacy, Lebanese International University, Beirut, Lebanon; 4 Faculty of Arts and Sciences, Holy Spirit University of Kaslik (USEK), Jounieh, Lebanon; 5 Research and Psychology Departments, Psychiatric Hospital of the Cross, Jal Eddib, Lebanon; Iwate Medical University, JAPAN

## Abstract

**Objectives:**

Nomophobia, an abbreviation of “No mobile phone phobia”, is characterized by the illogical fear of being detached from the mobile phone or unable to use it. Research have provided evidence of an association between increased cellular phone use and multiple health issues, such as anxiety, depression, insomnia, and others. To our knowledge, there are no Lebanese studies about nomophobia, despite the high incorporation rate of mobile phones in Lebanon and the likelihood of suffering from anxiety, depression, and other conditions due to nomophobic attitudes. The study objectives were to validate and confirm psychometric properties of the Nomophobia Questionnaire (NMP-Q) and examine the associations between particular psychological conditions (anxiety, depression, stress, insomnia and impulsivity) and nomophobia among a representative sample of Lebanese people.

**Methods:**

This cross-sectional study was carried out between January and July 2019. It enrolled 2260 residents of the community randomly selected from Lebanon’s Mohafazat. Two villages per sub-district and households from each village were chosen using a random sampling technique. A questionnaire was distributed randomly to the households. SPSS version 25 was used to perform the statistical analysis. A multinomial regression was computed taking the nomophobia categories as the dependent variable (and taking the absence of nomophobia as the reference category) and all variables that showed a significant association in the bivariate analysis as independent variables.

**Results:**

A total of 2260 (80.71%) out of 2800 questionnaires distributed was collected back. The mean age of the participants was 27.98 ± 9.66 years (58.8% females). Moreover, the mean nomophobia score was 71.56 ± 26.92 (median = 71; minimum = 14; maximum = 140). The results showed that 46 (2.0%) had no nomophobia, 769 (34.1%) mild nomophobia [95% CI 0.322–0.361], 1089 (48.3%) moderate nomophobia [95% CI 0.463–0.504] and 349 (15.5%) severe nomophobia [95% CI 0.140–0.170]. Items of the nomophobia scale converged over a solution of three factors that had an Eigenvalue over 1 (Factor 1 = emotions associated to losing connectedness, Factor 2 = not being able to communicate, Factor 3 = not being able to access information; total variance explained = 66.65%, and Cronbach’s alpha = 0.948). The results of a multinomial regression, taking the nomophobia score as the dependent variable, showed that higher age was significantly associated with lower odds of having mild (aOR = 0.97), moderate (aOR = 0.93) and severe (aOR = 0.97) nomophobia respectively. Higher anxiety (aOR = 1.09) and higher insomnia (aOR = 1.04) were significantly associated with higher odds of having severe nomophobia.

**Conclusion:**

The results suggest a positive correlation between nomophobia and psychological conditions. There is a need for longitudinal and prospective studies that furnish information with regards of the impact of time on the variables measured, in order to better understand the nature, causes, and attributes of nomophobia.

## Introduction

A challenging modern-day phobia, called nomophobia, is on the rise. Recently, the mass media and the professionals in the fields of psychology have been giving a peculiar attention to this emerging condition, not yet considered as a disease in the official manuals of psychiatric diagnoses. Nomophobia, an abbreviation of “No mobile phone phobia”, is characterized by the illogical fear of being detached from the mobile phone or unable to use it [1]. The term “Nomophobia” first appeared in a 2008 United Kingdom (UK) Post Office study conducted by YouGov, a UK research organization, which evaluated anxiety in mobile phone users [2]. Individuals suffering from nomophobia experience nervousness induced by losing their mobile phones, not having connection signal, and with batteries running out of power. They exhibit a growing preference for communication through technological devices, rather than a face-to-face direct communication with individuals, and feel secure when receiving contact through their cell phones [3].

Despite the fact that research provides no major information concerning the underlying causes of nomophobia, few factors have been implicated in this disorder. As conceptualized by Bianchi and Philips, nomophobia involves multiple psychological causative factors. In fact, few studies have highlighted a form of social anxiety related to social networks, called “FOMO” or the “Fear of missing out”, described by the necessity of being constantly online, and that could lead to nomophobia [4]. Along with that, extrovert and perfectionist personalities could potentially display an obsessive preoccupation with being present on social events, and a consequent fear of being separated from their mobile devices [2].

Further, research have provided evidence of an association between increased cellular phone use and multiple health issues, such as anxiety, depression, insomnia, and others [5]. It has been reported that excessive mobile phone users often experience episodes of anxiety when being in poor network zones, or when, accidentally, their phone credits runs out [6]. Moreover, a study conducted among Hong Kong secondary school students showed a positive correlation between problematic mobile phone usage and daytime sleepiness, as well as depression [7]. Individuals with nomophobia, continuously checking for any notification, develop the urge to sleep with their mobile phones. The results of a Japanese study analyzing sleep patterns in mobile phone users after lights out revealed poor quality of sleep, less total sleeping hours and insomnia in the studied population [8, 9]. What is more, nomophobia is often coupled with problems in controlling impulse and inability to delay satisfaction, concerning new functionalities incorporated in mobile phones. Eventually, the attitudes driven by seeking originality tend to increase impulsivity and lose self-discipline [10, 11]. The study of post office in UK compared stress levels induced by nomophobia and found a relatively high percentage in men than in women [12].

**Fig 1:**
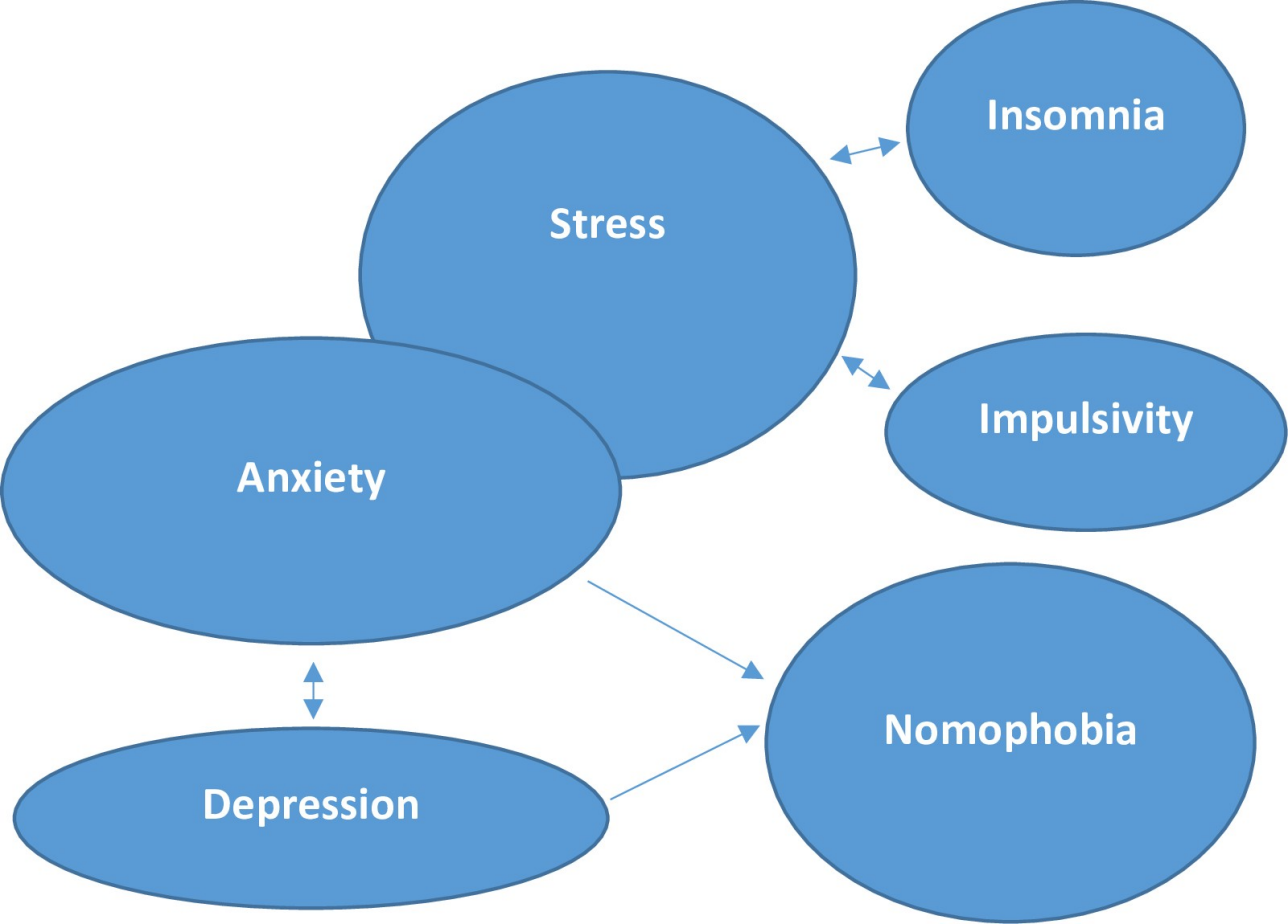
Descriptive graphic in the contexts of nomophobia, anxiety, depression, stress, insomnia and impulsivity.

Our theoretical framework focuses on integrating the concepts of nomophobia, anxiety, depression, stress, insomnia as well as impulsivity based on different studies associating theses variables together (Fig 1) [13–16].

Nomophobia lacks ample literature and has obtained very little empiric attention, with no study having completely revised its diagnostic criteria. The prevalence rates of nomophobia were similar between different studies, with most of the scores being within the moderate range of nomophobia [17]. De facto, two studies concerning college students reported that approximately 60% had moderate nomophobia, whereas severe and mild nomophobia scored 25% and 15% respectively [18]. As clearly stated by plenty of studies, the only instrument that assesses nomophobia with robust psychometric testing is the 20-item Nomophobia Questionnaire (NMP-Q) [19]. It has been validated in different languages such as in the Italian after translation from English version [20], Chinese [21] and Portuguese [22]. Notably, the Arabic version of NMP-Q demonstrated satisfactory validity, as proven in a study realized in Kuwait [23] and Morocco [24], but there is no validation among Lebanese population.

Investigating whether any relationship exists between nomophobia and specific psychological conditions (anxiety, depression, distress, as well as insomnia and impulsivity) will be an addition and an enforcement to the ongoing prevention and intervention efforts. With technology continuing to govern most facets of individual’s lives, nomophobia could be the next epidemic where a person is physically present but psychologically absent. The possible detrimental effect of nomophobia on mental health makes of this entity a highly important topic to study in order to reach preventive measures and possible therapies. To our knowledge, there are no Lebanese studies about nomophobia, despite the high incorporation rate of mobile phones in Lebanon and the likelihood of suffering from anxiety, depression, and other conditions due to nomophobic attitudes [25]. For those reasons, our study aims to validate and confirm psychometric properties of the Nomophobia Questionnaire (NMP-Q) and to study the associations between particular psychological conditions (anxiety, depression, stress, insomnia and impulsivity) and nomophobia among a representative sample of Lebanese people.

## Methods

### Ethical approval and consent to participate

The Psychiatric Hospital of the Cross ethics committee approved the study protocol (reference: HPC-21-2019). A written informed consent was obtained from each participant.

### Study design and participants

From the period of January until July 2019, we conducted a cross-sectional study targeting all Lebanese districts. The latter are divided into sub-districts, which are divided into villages. Two villages per sub-district were chosen via a random sampling technique. Households from each village were chosen using the same sampling technique [26]. Individuals from one household aged more than 18 years and who agreed to take part in the study were requested to fill the survey through a face-to-face interview. Individuals with cognitive impairment and those who declined participation were excluded. Study-independent personnel performed the collection of data.

### Minimal sample size calculation

Based on the formula $n=\frac{{\left(Z_{1-\alpha /2}\right)}^{2}p(1-p)}{d^{2}}$, where n = size of the sample, p = expected proportion and d = the desired margin of error and *Z*_1−*α*/2_ = 1.96 for *α* = 5%, a minimal sample of 1152 participants was needed, based on a p = 50% expected frequency of nomophobia in the absence of similar studies, a d = 5% risk of error and a design effect of 3.

### Questionnaire

The survey used during the interview was in Arabic, the native language of Lebanon. The questionnaire consisted of two parts. Part one included the sociodemographic characteristics of the participants (age, gender, parents’ status (living together or divorced) and the house crowding index; the latter reflects the socioeconomic status of the family and is calculated by dividing the number of persons living in the house by the number of rooms in the house besides the kitchen and bathrooms; a higher house crowding index indicates a lower socioeconomic status [27]). Part two consisted of scales/measures used in this study:

#### Nomophobia Questionnaire (NMP-Q)

It is a 20-item scale [23] that is scored based on a seven-point Likert scale, with 1 = do not agree at all and 7 = strongly agree. Higher scores indicate higher nomophobia. The total score yielded four categories of participants that had no nomophobia (scores of 20), mild nomophobia (scores between 21 and 59), moderate nomophobia (scores between 60 and 99) and severe nomophobia (scores between 100 and 140). The pre-existing Arabic version of the scale was used in this study [24]. The Cronbach’s alpha for this scale was excellent (α = 0.948).

#### Hamilton Anxiety Scale (HAM-A)

The HAM-A [28], recently validated in Lebanon [29], consists of 14 items, with higher scores reflecting more anxiety (in this study α_Cronbach_ = 0.949).

#### Hamilton Depression Rating Scale (HDRS)

The validated Arabic version of the HDRS was used in this study [30], with higher scores indicated more depression [31] (in this study α_Cronbach_ = 0.895).

#### Beirut Distress Scale (BDS-10 scale)

This scale validated in Lebanon is used to measure the level of stress over the past week [32] and consists of 10 questions. Higher scores indicate higher stress levels (in this study α_Cronbach_ = 0.956).

#### Lebanese Insomnia Scale (LIS-18)

This 18-item scale was generated in Lebanon for insomnia screening [33]. Higher scores reflect higher insomnia (in this study α_Cronbach_ = 0.762).

#### Barratt Impulsiveness Scale (BIS-11)

It is a self-rated scale containing 30 items, with higher scores reflecting higher impulsiveness [34] (in this study α_Cronbach_ = 0.814).

### Forward and back translation procedure

A psychologist performed the forward translation of the BIS-11 (from English to Arabic). A committee of expert healthcare professionals (two psychologists and one psychiatrist) verified this translation. The backward translation (From Arabic to English) was done by another psychologist. Then the committee matched the back-translated English questionnaire with the original scales version to detect inconsistencies. Discrepancies were resolved by consensus.

### Statistical analysis

SPSS version 25 was used to perform the statistical analysis. The person who performed data entry was not involved in the data collection process. Weighting to the general population was performed regarding gender, age, and governorate of dwelling; weighting factors are used to make samples match the population and to make it as representative as possible. Two different methods were used to confirm the nomophobia questionnaire construct validity. First, a factor analysis was run on sample 1. Since the extracted factors were found to be significantly correlated, the Promax rotation technique was used. To ensure the model’s adequacy, the Kaiser-Meyer-Olkin (KMO) measure of sampling adequacy and Bartlett’s test of sphericity were calculated. Factors with an Eigen value higher than one were retained. Moreover, Cronbach’s alpha was recorded for reliability analysis for the total scale and its subscales. Second, a confirmatory factor analysis was carried out on Sample 2 using the Statistica software. To assess the structure of the instrument the maximum likelihood method for discrepancy function was used. Several goodness-of-fit indicators were reported: Relative chi square (x2/df), Root Mean Square Error of Approximation (RMSEA), Goodness of Fit Index (GFI) and the Adjusted Goodness of Fit Index (AGFI). The index of goodness of fit was calculated by the value of x2 divided by the degrees of freedom (x2/df) (cut-off values <2–5). The RMSEA tests the fit of the model to the covariance matrix. As a guideline, values of <0.05 indicate a close fit and values below 0.11 an acceptable fit. The GFI and AGFI are chi-square-based calculations independent of degrees of freedom. The recommended thresholds for acceptable values are ≥0.90 [35].

A bivariate analysis was conducted using the Chi-square test to check for associations between the nomophobia categories and dichotomous variables (i.e. gender), and the ANOVA test to compare means of continuous variables and the nomophobia categories. Thereafter, we performed multivariable analyses; a generalized linear model (GLM) was conducted taking the continuous nomophobia score as the dependent variable, whereas a multinomial regression was computed taking the nomophobia categories as the dependent variable (and taking the absence of nomophobia as the reference category). All variables that showed a significant association in the bivariate analysis as independent variables. Finally, to examine the structural relationship between the before mentioned variables (insomnia, impulsivity, depression, anxiety and stress) and nomophobia, a Structural equation modeling (SEM) was performed using SPSS AMOS v24. The goodness-of-fit of the model was verified by the RMSEA and the comparative fit index (CFI), the most frequently used indices [36]. Values of RMSEA ≤0.06 and CFI values >0.90 indicate good fit of the model [36]. P<0.05 was considered statistically significant.

## Results

A total of 2260 (80.71%) out of 2800 questionnaires distributed was collected back. The mean age of the participants was 27.98 ± 9.66 years (58.8% females). In addition, 380 (16.8%) had divorced parents. The mean household crowding index was 1.07 ± 0.48. Moreover, the mean nomophobia score was 71.56 ± 26.92 (median = 71; minimum = 14; maximum = 140). Also, the results showed that 46 (2.0%) had no nomophobia, 769 (34.1%) mild nomophobia, 1089 (48.3%) moderate nomophobia and 349 (15.5%) severe nomophobia.

### Factor analysis

Out of all the items of the nomophobia scale, no item was removed. Half of the original sample has conducted the exploratory factor analysis (Total n = 1130). Items converged over a solution of three factors that had an Eigenvalue over 1 (Factor 1 = emotions associated to losing connectedness, Factor 2 = not being able to communicate, Factor 3 = not being able to access information; total variance explained = 66.65%, KMO = 0.951, Bartlett’s test of sphericity p<0.001 and Cronbach’s alpha = 0.948). According to the Promax rotated matrix, the components are summarized in Table 1.

**Table 1:** Promax rotated matrix of the nomophobia scale items using a forced three-factor solution.

Items	Item number	Factor Loading			
		1	2		3
**Factor 1: Emotions associated to losing connectedness** (α = 0.824)					
I would feel awkward because I could not check my notifications for updates from my connections and online networks	18	0.840			
I would feel weird because I would not know what to do	20	0.807			
I would be uncomfortable because I could not stay up-to-date with social media and online networks	17	0.802			
I would feel anxious because I could not check my email messages	19	0.785			
If I were to run out of credits or hit my monthly data limit, I would panic	6	0.749			
I would be nervous because I would be disconnected from my online identity	16	0.696			
If I could not check my smartphone for a while, I would feel a desire to check it	9	0.648			
Running out of battery in my smartphone would scare me	5	0.614			
I would be nervous because I could not know if someone had tried to get a hold of me	14	0.553			
If I did not have a data signal or could not connect to Wi-Fi, then I would constantly check to see if I had a signal or could find a Wi-Fi network	7	0.518			
If I could not use my smartphone, I would be afraid of getting stranded somewhere	8	0.439			
**Factor 2**: **Not being able to communicate** (α = 0.908)					
I would be worried because my family and/or friends could not reach me	11			0.935	
I would feel anxious because I could not instantly communicate with my family and/or friends	10			0.811	
I would be anxious because I could not keep in touch with my family and/or friends	13			0.803	
I would feel anxious because my constant connection to my family and friends would be broken	15			0.686	
I would feel nervous because I would not be able to receive text messages and calls	12			0.542	
**Factor 3 Not being able to access information** (α = 0.925)					
I would feel uncomfortable without constant access to information through my smartphone	1				0.846
I would be annoyed if I could not look information up on my smartphone when I wanted to do so	2				0.837
Being unable to get the news (e.g., happenings, weather, etc.) on my smartphone would make me nervous	3				0.640
I would be annoyed if I could not use my smartphone and/or its capabilities when I wanted to do so	4				0.640
**Percentage of variances explained**		32.68		8.27	7.70

### Confirmatory factor analysis on sample 2

Using the same EFA solution obtained in sample 1, we conducted a confirmatory factor analysis on sample 2. The results were as follows: The Maximum Likelihood Chi-Square = 783 and Degrees of Freedom = 310, which gave an x2/df = 2.52. For non-centrality fit indices, the Steiger-Lind RMSEA was on 0.134 [0.113–0.164]. Moreover, the Joreskog GFI equaled 0.758 and AGFI equaled 0.778.

### Bivariate analysis

The results of the bivariate analysis are summarized in Table 2. A higher percentage of females had moderate nomophobia, whereas a higher percentage of participants whose parents live together had severe nomophobia. Higher anxiety, depression, insomnia and impulsivity were significantly found in participants with severe nomophobia. Higher stress was significantly found in participants with moderate nomophobia, whereas higher age and BMI were significantly found in those with no nomophobia.

**Table 2:** Bivariate analysis of factors associated with nomophobia.

Variable	Nomophobia score				*p*
	No nomophobia	Mild nomophobia	Moderate nomophobia	Severe nomophobia	
**Gender**					**0.004**
Male	20 (44.4%)	354 (46.3%)	414 (38.2%)	135 (38.7%)	
Female	25 (55.6%)	410 (53.7%)	671 (61.8%)	214 (61.3%)	
**Parental status**					0.128
Living together	33 (71.7%)	643 (83.6%)	903 (82.9%)	298 (85.4%)	
Separated	13 (28.3%)	126 (16.4%)	186 (17.1%)	51 (14.6%)	
**Impulsivity**	72.37 ± 7.12	71.81 ± 7.15	72.87 ± 6.34	74.25 ± 7.46	**<0.001**
**Self-esteem**	29.06 ± 6.18	29.36 ± 4.49	29.21 ± 4.37	29.23 ± 5.06	0.787
**Stress**	6.24 ± 9.53	10.15 ± 11.20	12.40 ± 13.58	11.88 ± 13.86	**0.003**
**Anxiety**	3.56 ± 7.37	7.12 ± 9.19	9.93 ± 11.15	11.30 ± 12.94	**<0.001**
**Depression**	3.24 ± 5.07	5.88 ± 7.60	7.51 ± 8.74	7.63 ± 8.89	**<0.001**
**Insomnia**	34.29 ± 19.70	39.14 ± 19.31	40.32 ± 19.80	40.99 ± 20.20	**<0.001**
**Age**	33.65 ± 12.10	30.86 ± 12.87	26.00 ± 10.20	26.72 ± 10.87	**<0.001**
**House crowding index**	0.92 ± 0.34	1.06 ± 0.46	1.08 ± 0.49	1.09 ± 0.47	0.238
**Body Mass Index**	25.68 ± 3.10	24.66 ± 4.56	24.35 ± 10.06	24.56 ± 4.54	**<0.001**

Numbers in bold indicate significant p-values.

### Multivariable analysis

The results of multinomial logistic regression, taking the nomophobia categories as the dependent variable (absence of nomophobia taken as the reference category), showed that higher age (aOR = 0.97) was significantly associated with lower odds of having mild nomophobia compared to no nomophobia (Table 3, Model 1). Higher age (aOR = 0.93) was significantly associated with lower odds of having moderate nomophobia compared to no nomophobia (Table 3, Model 2). Higher age (aOR = 0.94) was significantly associated with lower odds of having severe nomophobia compared to no nomophobia. Higher anxiety (aOR = 1.09) and higher insomnia (aOR = 1.04) were significantly associated with higher odds of having severe nomophobia compared to no nomophobia (Table 3, Model 3).

**Table 3:** Multinomial logistic regression taking the nomophobia categories as the dependent variable.

	*p*	aOR	95% Confidence Interval	
			Lower bound	Upper bound
Model 1: Mild nomophobia vs absence of nomophobia*				
Age	**0.033**	0.97	0.94	0.99
Model 2: Moderate nomophobia vs absence of nomophobia*				
Age	**<0.001**	0.93	0.91	0.96
Model 3: Severe nomophobia vs absence of nomophobia*				
Age	**<0.001**	0.94	0.91	0.97
Anxiety	**0.023**	1.09	1.01	1.17
Insomnia	**0.048**	1.04	1.01	1.08

*Reference group; Pseudo R-square Nagelkerke = 11.6%; Goodness of fit Pearson value = 4661.67 (p<0.001).

The results of the Generalized Linear Model (GLM) taking the continuous nomophobia score as the dependent variable, showed that higher impulsiveness (β = 0.31), higher anxiety (β = 0.44), and higher insomnia (β = 0.22) were significantly associated with higher nomophobia scores, whereas higher stress (β = -0.15) and higher age (β = -0.44) were significantly associated with lower nomophobia scores (Table 4).

**Table 4:** Generalized Linear Model (GLM) taking the continuous nomophobia score as the dependent variable.

Variable	Beta	*p*	95% Confidence Interval	
Impulsiveness	0.31	**0.003**	0.11	0.52
Stress	-0.15	**0.036**	-0.28	-0.01
Anxiety	0.44	**<0.001**	0.28	0.59
Depression	0.05	0.669	-0.16	0.25
Body Mass Index	0.02	0.781	-0.14	0.18
Age	-0.44	**<0.001**	-0.56	-0.32
Insomnia	0.22	**0.001**	0.09	0.36
Gender (males compared to females*)	-1.23	0.396	-4.06	1.61

*Reference group; Goodness of fit value = 977854.21; numbers in bold indicate significant p-values.

### Path analysis- structural equation modeling

**Fig 2:**
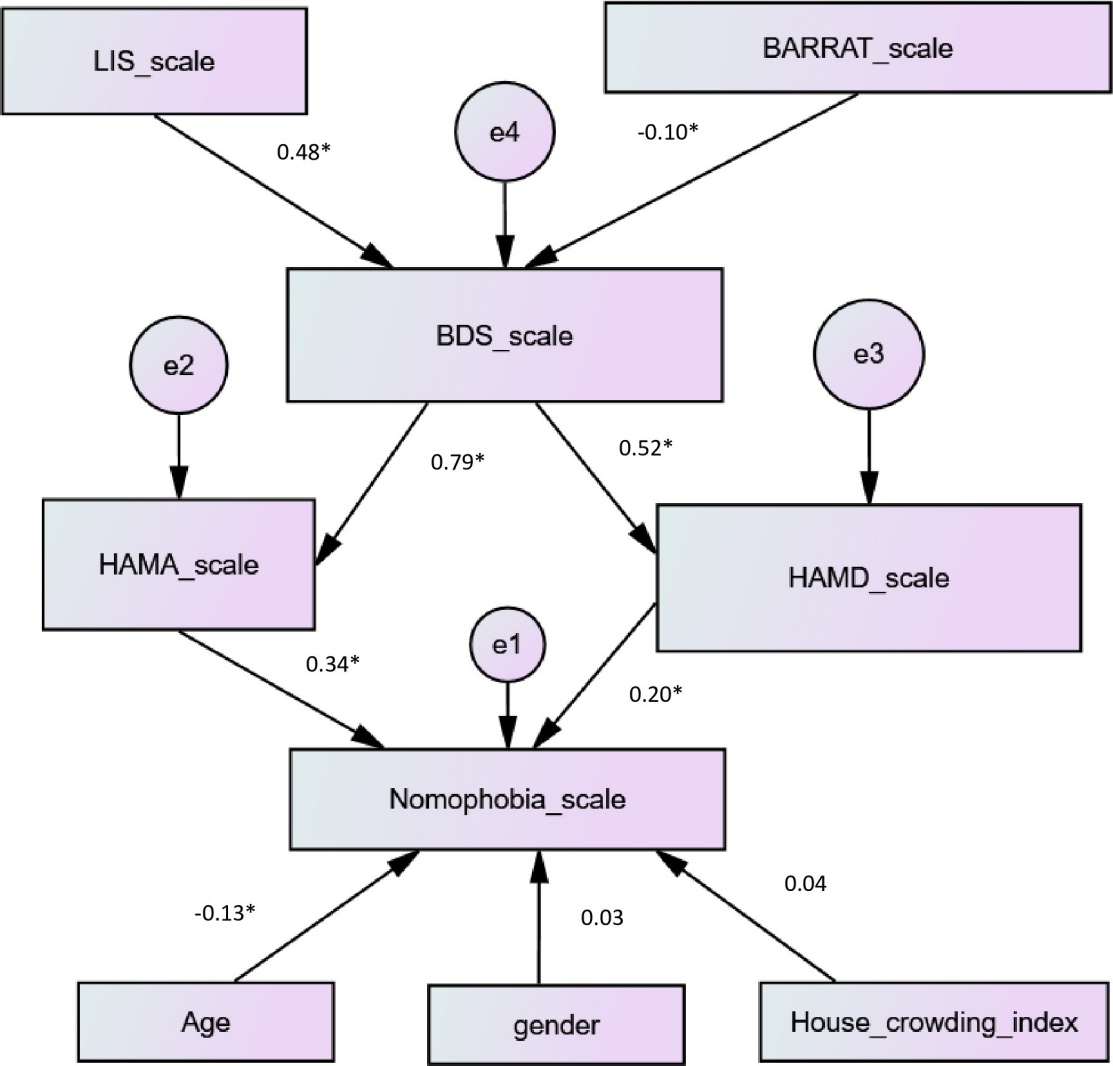
Structural equation modeling of the variables associated with nomophobia. 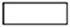
—observed variable; 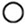
—latent variable; 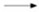
—impact of one variable on another; e—residual error in the prediction of an unobserved factor; * p<0.001; BDS = Beirut Distress Scale; HAM-A = Hamilton Anxiety Scale; HAM-D = Hamilton Depression Scale; LIS = Lebanese Insomnia Scale; BARRAT scale used to measure impulsiveness.

Fig 2 summarizes the SEM of factors associated with nomophobia. The model’s fit was verified (RMSEA = 0.043; PCLOSE = 0.552; CFI = 0.96). The path coefficients for the path from insomnia to stress, from impulsivity to stress, from stress to anxiety, from stress to depression, from age to nomophobia, from anxiety to nomophobia and from depression to nomophobia were all highly significant (p<0.001 for all) (Fig 2).

## Discussion

By this time, our project is the first to evaluate the association between particular psychological conditions and nomophobia on the Lebanese population. Taking into account the results obtained in different studies, nomophobia could exhibit negative influence on mental health [37]. Therefore, we found quite intriguing to assess nomophobic attitudes in a country that is witnessing high levels of smartphones dependence [38]. The results showed that higher anxiety and insomnia were associated with greater nomophobia scores, whereas higher age was associated with lower nomophobia scores. This implies links between nomophobia and the studied factors, which are worthwhile investigating for a better understanding of this relatively new phobia.

The internal consistency for all NMP-Q items and the 3 subscales was excellent, showing that this scale is capable of producing consistent results. Our results were similar to those of the original version of the questionnaire (αCronbach = .945 for the total score and between .819 and .939 for the subscales) [1]. The three-factor solution obtained in the current Arabic version of the NMP-Q (Factor 1 = emotions associated to losing connectedness, Factor 2 = not being able to communicate, Factor 3 = not being able to access information) was comparable to the four-factor solution obtained by previous authors [1]. Accordingly, using the NMP-Q to assess nomophobia among Lebanese population is recommended.

Varying, but significant degrees of nomophobia prevalence were found among the participants. In point of fact, our results showed that 34.1% had mild nomophobia, 48.3% moderate nomophobia and 15.5% severe nomophobia, while the mean nomophobia score was 71.56 ± 26.92. Up today, there is no study touching on nomophobia in Lebanon. However, few ones were conducted in different countries and most of the nomophobia scores fell within the moderate range [18], similarly to our results. It is compelling to mention that nearly all of the participants in our study reported some degree of nomophobia (2% only had no nomophobia), which was discovered to be the same in the international studies as well.

The level of anxiety showed significant correlation with nomophobia. Comparable conclusions were shown in a study by Veerapu et al. where nomophobia anxiety were positively correlated [39]. Another study presenting a proposition for including nomophobia in the new Diagnostic and Statistical Manual of Mental Disorders (DSM–5) demonstrated that the consequences of nomophobia are more evident in subjects with existing ailments, notably anxiety, suggesting a particular pattern of connection between the different entities [40]. Furthermore, social anxiety appearance, a consequence of negative body image-related appearance, affected nomophobia levels according to a study carried out on nursing students in western Turkey [41]. Actually, nomophobic behaviors strengthen social anxiety tendencies, since it generates an addiction to virtual and digital communications, in order to relieve the daily social stress. In many occasions, this phobic attitude might present exaggerated actions pushing beyond the standards in terms of having a healthy lifestyle and subsequently inducing imbalance [2].

By the same tokens, the levels of insomnia showed significant correlation with nomophobia. In a study conducted among Japanese teenagers, the hours of usage of cellular phone was significantly associated with insomnia [42]. Back to Veerapu et al. once again, they also highlighted an association between sleeping difficulties and nomophobia [39]. Indeed, experiencing intense feelings of frustration and fear when unable to use mobile phone creates emotional and psychological alterations, which plainly explains the occurrence of insomnia in nomophobia cases. Moreover, sleep disturbances were dependent to a precise category of activity in which the person is engaged on the mobile phone. As a matter of fact, findings in the literature show that cellular gaming does not appear to be problematic neither affect sleep quality, contrarily to social network services and online chats [42, 43].

Further, as indicated by our outcomes, higher age was linked to lower levels of nomophobia. In opposite to our study, previous researchers confirm finding no relation between nomophobia and age [44–46], proving that nomophobia may appear at whatever stage in life [10]. The relationship between these two variables is still controversial, hence, more research is needed based on samples from all ages [14].

The analysis for the path from insomnia to stress, impulsivity to stress, stress to anxiety, stress to depression, anxiety to nomophobia and depression to nomophobia were all highly significant. In fact, Kales and Kales have documented that insomniacs are prejudiced to less satisfying interpersonal relationships, and have low self-concepts, which results in weak coping mechanisms for dealing with stress [47]. On the same level, Liu and Kleiman suggested that impulsive people who have a tendency to respond rashly to stimuli, rather than be engaged into effective negotiations are more likely to generate stressful events [48]. Moreover, as pointed out by our analysis, stress could lead to anxiety and depression. This finding was similar to a study in 2006 by Ardayfio and colleague which proved that repeated and recurrent stress quickens and worsens the mood disorders notably anxiety and depression [49]. Next in order, the latter two can precipitate nomophobic behaviors in agreement with other studies [50, 51]. In respect to that, our outcomes demonstrate the direct effect of anxiety and depression on nomophobia, as well as the indirect one they also carry on by mediating the relationship between stress and nomophobia.

### Clinical implications

This study procures various key contributions to academia [52]. The literature lacks of Lebanese studies discussing nomophobia. Since mobile phones penetration across total Lebanese population increased exceedingly from 36% in 2012 to 70% in 2014, according to a recent survey, it becomes important to assess the likelihood that smartphone use and addiction may engender nomophobia behaviors in the Lebanese population [38, 53]. This study also provides more understanding of nomophobia by evaluating psychological conditions and characteristics that could be related to it. Our results can help researchers assess, nomophobic tendencies and recognize its possible association.

### Limitations

The study is cross-sectional in nature, which may challenge the causal direction of the main associations. Nomophobia levels were evaluated adopting a questionnaire, and not through a clinical interview; for that reason, the responses may possibly bear some inaccuracies for not wanting to reveal vulnerabilities, or because of recall and information bias. Furthermore, the NMP-Q is not designed to make the diagnosis of nomophobia, it could only aid in the screening process of the disorder; a further assessment by a psychologist or a psychiatrist is necessary to make the definite diagnosis. A selection bias is also possible because of the refusal rate. A residual confounding bias is also possible since not all factors associated with nomophobia were taken into consideration in this study. Because only 2% of the participants had no nomophobia, our understanding of what underlies nomophobia becomes ambiguous as it could simply be the result of living in a digital world. This means that we could be talking about a normal condition rather than a pathological one. Moreover, more research is needed to prove whether nomophobia could happen independently from other anxiety disorders or is just a specific expression of generalized anxiety.

## Conclusion

The results suggest a positive correlation between nomophobia and psychological conditions. Although, the new DSM-5 has widened the criteria for addictive disorders to comprise certain non-substance behavioral addictions, defined criteria for nomophobia are not yet delineated. Some dimensions of this disorder are still not explicit, which indicates that it is a suitable context for research. Further cohort studies are needed to better understand the nature, causes, and attributes of nomophobia. In addition, nomophobia levels should be assessed employing instruments, the reliability and validity of which are established.

## Supporting information

S1 Table The Nomophobia questionnaire (NMP-Q) Arabic language. (DOCX)

## List of abbreviations

NMP-Q: Nomophobia Questionnaire
HDRS: Hamilton depression rating scale
HAM-A: Hamilton anxiety scale
BDS-22: Beirut Distress Scale
LIS-18: Lebanese Insomnia Scale
BIS-11: Barratt Impulsiveness Scale
