# Supplementary material for: Nomophobia in Lebanon: Scale validation and association with psychological aspects
Source: PLoS One. 2021 Apr 20;16(4):e0249890. doi: 10.1371/journal.pone.0249890 (PMC8057610; doi:10.1371/journal.pone.0249890)
Supplement: S1 Table — (DOCX) [file pone.0249890.s001.docx]

**The Nomophobia questionnaire (NMP-Q) Arabic language**

| لا أوافق أوافق بشدّة  على الإطلاق | | | | | | | **يرجى الإشارة إلى أي مدى توافق أو لا توافق على كل عبارة فيما يتعلق بهاتفك الذكي** |
| --- | --- | --- | --- | --- | --- | --- | --- |
| 7 | 6 | 5 | 4 | 3 | 2 | 1 |  |
|  |  |  |  |  |  |  | 1- أحس بعدم الارتياح عندما لا يحق لي الوصول لمعلومة ما من خلال هاتفي  الذكي |
|  |  |  |  |  |  |  | 2- أشعر بالملل إن لم أستطع أن أرى معلومات في هاتفي الذكي إن أردت ذلك |
|  |  |  |  |  |  |  | -3 عدم استطاعتي لمعرفة الأخبار( مثل الأحداث، الطقس ...) بهاتفي الذكي قد يصيبني بالغضب |
|  |  |  |  |  |  |  | -4 أشعر بالضجر إن لم أستطع استعمال هاتفي الذكي أو التطبيقات عندما أريد ذلك |
|  |  |  |  |  |  |  | -5 انتهاء بطارية هاتفي الذكي قد يصيبني بالرعب |
|  |  |  |  |  |  |  | -6 عندما أشعر باقتراب نهاية الاشتراك الشهري للأنترنت أو المكالمات في هاتفي الذكي قد يصيبني بالهلع |
|  |  |  |  |  |  |  | - إن لم يكن لدي إشارة بيانات أو اتصال بالواي فاي أصبح كل برهة أتفقد  هاتفي إن عادت الاشارة أم لا |
|  |  |  |  |  |  |  | -8 إن لم أستطع استعمال هاتفي الذكي أشعر بالخوف من أن أعلق في مكان ما |
|  |  |  |  |  |  |  | -9 إن لم أستطع تصفح هاتفي لبرهة أشعر أنني أود ذلك |
|  |  |  |  |  |  |  | **إذا لم يكن معي هاتفي النقال** |
|  |  |  |  |  |  |  | 10- أشعر بالهلع لأنه ليس باستطاعتي التواصل مع أهلي أو أصدقائي |
|  |  |  |  |  |  |  | 11- أشعر بالقلق لأن عائلتي أو أصدقائي لا يستطيعون الوصول إلي |
|  |  |  |  |  |  |  | 12- أشعر بالعصبية لأنني لا أستطيع تلقي رسائل نصية أو مكالمات |
|  |  |  |  |  |  |  | 13- أشعر بالهلع لأنه ليس في استطاعتي ليس في استطاعتي أن أبقى على اتصال مع عائلتي أو أصدقائي |
|  |  |  |  |  |  |  | 14- أشعر بالغضب لأته ليس في استطاعتي معرفة إن كان أحدهم يحاول الاهتمام بي |
|  |  |  |  |  |  |  | 15- أشعر بالهلع لأن تواصلي الدائم مع عائلتي أو أصدقائي سينقطع |
|  |  |  |  |  |  |  | 16- أشعر بالعصبية لأنني منقطع عن عالمي الافترابي |
|  |  |  |  |  |  |  | 17- أشعر بعدم الارتياح لعدم تمكني من البقاء على اطلاع على الأخبار و الشبكات الاجتماعية |
|  |  |  |  |  |  |  | 18- أشعر بالارتباك عندما لا أستطيع التحقق من الاشعارات الحديثة من شبكة الأنترنت |
|  |  |  |  |  |  |  | 19- أشعر بالقلق لأنني لا أستطيع الاطلاع على رسائلي الالكترونية |
|  |  |  |  |  |  |  | 20- أشعر بالغرابة لأنني لا أعرف ما يجب علي القيام به |
